# Precision Repetitive Transcranial Magnetic Stimulation Over the Left Parietal Cortex Improves Memory in Alzheimer’s Disease: A Randomized, Double-Blind, Sham-Controlled Study

**DOI:** 10.3389/fnagi.2021.693611

**Published:** 2021-06-29

**Authors:** Yanli Jia, Luoyi Xu, Kehua Yang, Yingchun Zhang, Xinghui Lv, Zhenwei Zhu, Zheli Chen, Yunlong Zhu, Lili Wei, Xia Li, Mincai Qian, Yuedi Shen, Weiming Hu, Wei Chen

**Affiliations:** ^1^Department of Psychiatry, Sir Run Run Shaw Hospital, Zhejiang University School of Medicine, Hangzhou, China; ^2^Third People’s Hospital of Huzhou, Huzhou, China; ^3^The Third Hospital of Quzhou, Quzhou, China; ^4^Department of Geriatric Psychiatry, Shanghai Mental Health Center, Shanghai Jiao Tong University School of Medicine, Shanghai, China; ^5^School of Medicine, Hangzhou Normal University, Hangzhou, China; ^6^Department of Psychology and Behavioral Sciences, Zhejiang University, Hangzhou, China; ^7^Key Laboratory of Medical Neurobiology of Zhejiang Province, Hangzhou, China

**Keywords:** precision rTMS, left parietal cortex, Alzheimer’s disease, memory, cognition

## Abstract

**Objective:**

We aim to study the effect of precision repetitive transcranial magnetic stimulation (rTMS) over the left parietal cortex on the memory and cognitive function in Alzheimer’s disease (AD).

**Methods:**

Based on the resting-state functional magnetic resonance imaging, the left parietal cortex site with the highest functional connectivity to the hippocampus was selected as the target of rTMS treatment. Sixty-nine AD patients were randomized to either rTMS or sham treatment (five sessions/week for a total of 10 sessions). The Mini-Mental State Examination (MMSE), 12-Word Philadelphia Verbal Learning Test (PVLT), and Clinical Dementia Rating (CDR) were assessed at baseline and after the last session.

**Results:**

After a 2-week treatment, compared to patients in the sham group, those in the rTMS group scored significantly higher on PVLT total score and its immediate recall subscale score. Moreover, in the rTMS group, there were significant improvements after the 2-week treatment, which were manifested in MMSE total score and its time orientation and recall subscale scores, as well as PVLT total score and its immediate recall and short delay recall subscale scores. In the sham group, the PVLT total score was significantly improved.

**Conclusion:**

The target site of the left parietal cortex can improve AD patients’ cognitive function, especially memory, providing a potential therapy.

## Introduction

Alzheimer’s disease (AD), the most common subtype of dementia among elderly adults (Sosa-Ortiz et al., 2012; Fargo et al., 2014), is generally characterized by memory decline (the core symptom), language dysfunctions, mood and personality changes, loss of spatial and temporal orientation, and behavioral derangements, leading to impaired functioning in individuals’ occupational and social domains (Lane et al., 2018). The global number of people with dementia was 4.38 million in 2016 (Nichols et al., 2019), which would be more than triple by 2050, according to the World Health Organization (WHO) (2017). Among them, individuals with AD accounted for 50–75% (Alzheimer’s Disease International, 2019). Therefore, the financial burden caused by AD would also continue to increase substantially (Winblad et al., 2016).

Up to now, AD is mainly treated by focusing on decelerating cognition decline using clinically approved medications, such as cholinesterase inhibitors, including donepezil, rivastigmine, and galantamine, for mild and moderate AD (Birks, 2006), and memantine, a medication antagonizing the *N*-methyl-D-aspartate receptor, for moderate and severe AD (McShane et al., 2019). However, these medications can only be used for symptomatic treatment, with limited efficacy, and cannot prevent disease course (Fargo et al., 2014; Anand et al., 2017; Lane et al., 2018), and not all patients can tolerate it. In clinical trials, about 29% of patients in the active treatment group withdrew from the trial due to side effects, which was significantly higher than that in the placebo group (Birks, 2006). As a consequence, exploring some non-pharmacological therapeutic strategies is highly demanding.

Repetitive transcranial magnetic stimulation (rTMS) is an alternative therapeutic method that can non-invasively stimulate the brain in a rhythmic form through electromagnetic induction, thereby modulating cortical excitability and neural activity (Rossi et al., 2009; Valero-Cabré et al., 2017). Low-frequency rTMS decreases neural excitability in targeted cortical areas, whereas high frequency increases neural excitability (Gangitano et al., 2002). In healthy participants, rTMS could serve as possible modulators of cognitive function. For instance, subthreshold TMS (50% and 60% motor thresholds) applied over the right dorsolateral prefrontal cortex effectively modulated cognitive function (Bashir et al., 2020). Studies also showed that rTMS enhanced cognitive and motor functions of healthy old adults (Zimerman and Hummel, 2010). In 2008, the US Food and Drug Administration (FDA) approved rTMS for 4–6 weeks to clinically treat medication-resistant major depression (FDA approval K061053). Currently, rTMS has achieved preliminary results in improving cognitive function in AD patients. Meta-analysis studies revealed that high-frequency rTMS treatment positively affected cognitive function and global impression (Cheng et al., 2018; Dong et al., 2018; Lin et al., 2019). Another recent meta-analysis also revealed that compared with sham brain stimulation, high-frequency rTMS had short-term and long-term (lasting to 1 month after treatment) positive effects on the general cognition of AD patients (Chu et al., 2021). More concretely, significant improvements were found on language, including the accuracy of action naming and auditory sentence comprehension (Cotelli et al., 2006, 2008, 2011; Zhao et al., 2017), verbal and non-verbal agility (Devi et al., 2014), memory (Haffen et al., 2012; Zhao et al., 2017; Chu et al., 2021), and attention (Eliasova et al., 2014).

Regarding memory improvement, verbal working memory was significantly improved after 10 Hz rTMS with 100% resting motor threshold applied over the left prefrontal cortex in healthy participants (Ohn et al., 2008). A randomized, double-blind, and sham-controlled trial indicated that after a 6-week 20 Hz rTMS treatment applied over brain areas of parietal P3/P4 and posterior temporal T5/T6 consistent to the electroencephalogram 10–20 system (one session per day and 5 days per week for a total of 30 sessions), AD patients’ memory in Montreal Cognitive Assessment increased significantly compared with their baseline, especially in patients with mild AD (Zhao et al., 2017). One case study using 10 Hz rTMS over the left prefrontal cortex (one session per day and 5 days per week for a total of 10 sessions) as an adjunct to AD treatment revealed that after 1 month of treatment, the patient performed better on episodic memory and speed processing tests (Haffen et al., 2012). Nevertheless, the studies focusing on memory improvement in AD patients are underrepresented. Moreover, most previous studies used comprehensive cognitive questionnaires to assess the efficacy, not specifically for memory, and reached inconsistent results (Liao et al., 2015; Cheng et al., 2018; Lin et al., 2019). Meanwhile, as we all know, memory decline is related to the hippocampus buried deep in the brain. However, the stimulus target is determined by the 5-cm rule, electroencephalogram 10–20 system, or magnetic resonance imaging navigation (Cheng et al., 2018), and the stimulus focality is limited only to the cerebral cortex (Thielscher and Kammer, 2004). It is difficult to directly stimulate the hippocampus or precisely stimulate it indirectly. Therefore, it is necessary to select a location in the cerebral cortex as the target of rTMS based on functional connectivity between the hippocampus and the cortex.

Although the hippocampus played a vital role in the process of memory encoding and retrieval (Battaglia et al., 2011), studies suggested that the interaction between brain regions underlying memory, rather than a single brain region such as the hippocampus, was the key to memory improvement (Kim et al., 2016), and interactions of the hippocampus with other locations in the cortical–hippocampal network were observed in a functional magnetic resonance imaging study (Spaniol et al., 2009). The parietal cortex, as a component of the cortical–hippocampal network, was related to the memory function, such as episodic memory and working memory (Berryhill, 2012), which was validated in several rTMS studies. In healthy participants, using rTMS to stimulate the lateral parietal target site, with the highest functional connectivity to the hippocampus, could modulate cortical–hippocampal networks and concomitantly manipulate associative memory (Wang et al., 2014). These findings could be reproduced. Using similar rTMS settings, Freedberg et al. (2019) found that hippocampal functional connectivity increased significantly and that this effect was specific to the hippocampal network. Moreover, theta-burst TMS treatment applied over the posterior inferior parietal cortex might also serve as a potential therapy for specifically manipulating the encoding of new associative memory without influencing memory retrieval (Tambini et al., 2018). In AD patients, the integrity of the parietal memory network including the inferior parietal lobule was disrupted (Greene and Killiany, 2010; Hu et al., 2019). After a 2-week rTMS treatment (1,640 pulses, 20 Hz, 100 MT, one session per day and 5 days per week) applied over the left lateral parietal cortex, AD patients performed better on visual recognition memory and clock drawing test, indicating that high-frequency rTMS treatment could improve cognition and modulate functional connectivity of the brain network (Velioglu et al., 2021). Evidence also showed that in prodromal AD patients, a 2-week 20 Hz rTMS treatment applied over the precuneus enhanced their episodic memory and neural activity (Koch et al., 2018). Besides, the left hemisphere was selected due to the known role of the left lateral parietal cortex in memory retrieval (Wagner et al., 2005). Based on these findings, we select the left parietal cortex as the target site, instead of the common dorsolateral prefrontal cortex used in most previous studies (Liao et al., 2015), to improve episodic memory in addition to general cognition.

Herein, we recruit patients for a 2-week 10 Hz rTMS or sham treatment and adapt some evaluation scales to assess their memory and cognition. We have hypothesized that after a 2-week 10 Hz rTMS treatment based on the functional connectivity of cortical–hippocampal networks, AD patients’ memory and cognition improved significantly compared to the baseline or sham group.

## Materials and Methods

### Study Design

The study involved a double-blind, randomized, and sham-controlled trial, which was approved by the Ethics Committee of Sir Run Run Shaw Hospital, School of Medicine, Zhejiang University (number: 20170228-1), and was registered on the National Medical Research Platform of China (number: MR-33-20-004217)^1^. The patients were randomly assigned to either rTMS or sham treatment. The rTMS group patients received daily treatment sessions for 2 weeks (one session per day and 5 days per week for a total of 10 sessions), while the sham group patients received regular sham management. Neuropsychological assessments were performed at baseline and immediately after a 2-week treatment. The primary outcomes were the differences in the 12-Word Philadelphia Verbal Learning Test (PVLT) scores between the groups and between pre- and post-treatment. The secondary outcomes were the differences in the Mini-Mental State Examination (MMSE) scores.

### Patients

A total of 103 patients with mild to moderate AD were recruited from outpatients and inpatients of the Sir Run Run Shaw Hospital, School of Medicine, Zhejiang University, Hangzhou, China, and underwent a standardized evaluation. The patients were screened from an ongoing follow-up project that aimed to treat AD by precision rTMS of the left parietal cortex. Patients were included if they (1) met the diagnostic criteria for probable AD according to the *Diagnostic and Statistical Manual of Mental Disorders*, fifth edition (DSM-5), criteria (American Psychiatric Association, 2013); (2) had a Clinical Dementia Rating (CDR) score between 0.5 and 2; and (3) were from 55 to 85 years old and right-handed. The exclusion criteria included: (1) severe liver, kidney, heart, and lung diseases; (2) history of head trauma, neurological disorders, psychiatric disorders, and/or substance abuse; (3) focal brain lesions on T1 or T2 images; (4) any rTMS contraindications (e.g., medical implants or devices, metal in the body, or epilepsies). Besides, for patients who used medications stably for more than 3 months, we continued the same medication types and doses during the 2-week rTMS sessions. All patients signed an informed consent form before the administration of the baseline assessment.

The patients were randomly assigned to groups with a single random number sequence that was used to produce a series of opaque and sealed envelopes. The envelope for each patient was opened by the rTMS therapist before the first treatment session.

### MRI Data Acquisition

T1-weighted and resting-state functional magnetic resonance imaging (fMRI) data were acquired using a 3.0-T Siemens Prisma MRI scanner (GE Discovery MR750, GE Medical Systems, Milwaukee, WI, United States) equipped with an eight-channel head coil array. During scanning, all patients were instructed to keep their head and body motionless with their eyes open and not think about anything specific. The functional images were obtained axially using an echo-planar imaging (EPI) sequence with the following parameters: 240 volumes; repetition time (TR) = 2,000 ms; echo time (TE) = 30 ms; flip angle (FA) = 90°; field of view (FOV) = 220 mm × 220 mm; matrix = 64 × 64; and slice thickness = 3.2 mm with no gap. High-resolution anatomic three-dimensional T1-weighted images were obtained with the following parameters: 176 axial slices; TR = 8.1 ms; TE = 3.1 ms; FA = 8°; FOV = 250 mm × 250 mm; matrix = 250 × 250; and slice thickness = 1.0 mm with no gap.

### rTMS Procedures

Each patient’s stimulus target was precisely identified using personal maps of hippocampal resting-state functional connectivity obtained at baseline. According to the method developed by Wang et al. (2014), based on the personal map, the average hippocampus coordinate [Montreal Neurological Institute (MNI) coordinates: *x* = −24, *y* = −18, *z* = −18] was selected as the seed of whole-brain functional connection analysis to identify the left parietal site with the highest functional connectivity to the hippocampus. This is the local highest connectivity within a 15-mm radius of MNI coordinates *x* = −47, *y* = −68, *z* = + 36 [an area including the inferior parietal lobule (supramarginal and angular gyrus) and Brodmann areas 39 and 40], and this site was designated as the target of rTMS treatment.

We applied rTMS treatment over the left lateral parietal site, guided by an online neuronavigation system (Brainsight 2, Rogue Research, Montreal, QC, Canada). The patients received 10 Hz rTMS treatments for 2 weeks (one session per day and 5 days per week for a total of 10 sessions). In every rTMS session, the patient was seated in a reclining armchair and was asked to keep the head still. The figure-of-eight coil (70-mm diameter) connected to a Magstim Rapid2 stimulator (Magstim Company, Whitland, Wales, United Kingdom) was held tangential to the target site with reference to a frameless infrared stereotactic system. The motor threshold was defined as the minimum TMS intensity that produced a motor evoked potential of at least 50 μV for at least 5 of 10 consecutive pulses at baseline. We determined the resting motor threshold by stimulating the left motor cortex, which required at least 5 of 10 consecutive pulses that must evoke contraction in the first dorsal interosseous muscle of the right hand when the patients kept the first dorsal interosseous muscles relaxed in both hands (Rossini et al., 2015). Each treatment included 800 pulses at 10 Hz and 100–110% motor threshold, and the stimulation lasted for 2 s, followed by a 28-s stimulation interval. The sham group received regular sham management such that the same coil was rotated 45° away from the brain scalp, with one wing of the coil being in contact with the scalp and the distance between the coil center and the target site being larger than 5 cm, and the patients would also perceive the same noise and sensation caused by the stimulation.

### Blinding

Sham treatment produces the same noise and sensation, and patients were told that scalp discomfort and transient fatigability might occur during rTMS or sham sessions. All patients and neuropsychologists administering clinical assessments did not know whether the patients received rTMS or sham treatment; only the rTMS therapist knew the randomized treatment. After every treatment session, patients were asked how they felt about the treatment to confirm that they did not know which treatment they received.

### Neuropsychological Assessments

All patients underwent several clinical assessments administered by a trained neuropsychologist at baseline and after the last (10th) session, including the following evaluation scales: the MMSE (Folstein et al., 1975); a brief screening questionnaire assessing cognitive impairment, including orientation, registration, recall, attention and calculation, and language ability to follow spoken and written commands; the PVLT (Bezdicek et al., 2014), a learning task consisting of three categories of words used to assess immediate, short delay, long delay, and distractor memory in AD patients; and the CDR (Hughes et al., 1982), a semi-structured diagnostic interview for the staging of clinical dementia in the elderly.

The primary outcome measure was the difference of the PVLT scores between the rTMS group and sham group and that between the baseline and post-treatment in both groups. The secondary outcome measure was the difference of the MMSE scores between the rTMS group and sham group and that between the baseline and post-treatment in both groups.

### Sample Size and Power Analysis

In our pre-experiment, the mean increment in PVLT score from baseline to 2 weeks was 6.10 points (SD = 4.80) in the rTMS group and 1.80 points (SD = 3.58) in the sham group. A relatively conservative difference of 4.00 points (SD = 5.00) between the two groups was used to estimate the sample size. The statistical analysis showed that a total of 68 patients (34 per group) were sufficient to provide a power of approximately 90% at a 5% significance level. Considering the potential study dropout, the sample size was increased by 25%, to 43 patients per group.

### Statistical Analyses

The statistics program SPSS version 22.0 (SPSS Inc., Chicago, IL, United States) was employed in this study, and all data were checked for normal distribution *p* > 0.05 in the Shapiro–Wilk test. Descriptive statistics for demographics and baseline measures in the two groups of participants were compared using Student’s *t*-test or a non-parametric Mann–Whitney test for continuous data or a χ^2^ test for categorical data. Within each group, a paired-sample two-tailed *t*-test was used to compare the mean scores at baseline and after the last (10th) session, and a Wilcoxon signed-rank test was utilized for non-parametric scores. Two-way ANOVA (group × scale) was applied to the mean scores after 2 weeks of 10 sessions between the two groups. Whenever a significant main effect was found, *post hoc* Student’s *t*-test was employed for comparison. Meanwhile, repeated-measures ANOVA, using group as the between-group factor and time as the within-group factor, was performed to assess the effect of rTMS intervention. Two-sided *p*-values less than 0.05 were considered significant.

## Results

**FIGURE 1:**
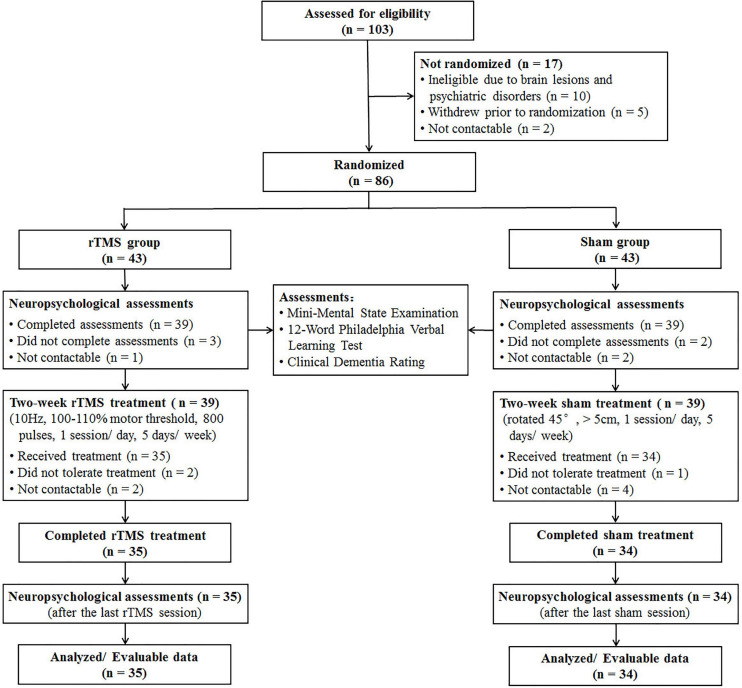
CONSORT diagram of patients with Alzheimer’s disease in a double-blind, sham-controlled study of rTMS.

Screening, enrollment, and participation information are shown in Figure 1. Before randomization, 10 patients were excluded due to brain lesions and psychiatric disorders, five patients withdrew due to personal reasons, and two patients were not contactable. After randomization, eight patients in the rTMS group and nine patients in the sham group did not complete neuropsychological assessments at baseline or did not tolerate the rTMS/sham treatment due to adverse effects, i.e., scalp discomfort and transient fatigability.

### Patients’ Baseline Characteristics

A total of 69 patients completed the 2-week treatment, and 35 of them were assigned to the rTMS group, and others were assigned to the sham group. No significant differences were found between the two groups as to gender (χ^2^ = 0.12, *p* = 0.733), age (*t* = -0.99, *p* = 0.325), education level (*t* = 0.15, *p* = 0.878), medication using (χ^2^ = 0.12, *p* = 0.726), and neuropsychological assessments (MMSE, PVLT, and CDR scores, see Table 1).

**TABLE 1:** Baseline characteristics of AD patients.

**Characteristics**	**rTMS group (*n* = 35)**	**Sham group (*n* = 34)**	***p*-value**
Age (years)	71.41 ± 8.85	73.41 ± 7.73	0.325
Female (%)	25 (71.43%)	23 (67.65%)	0.733
Education (years)	7.70 ± 5.26	7.50 ± 5.19	0.878
Medication (%)	20 (57.14%)	18 (52.94%)	0.726
MMSE	15.71 ± 5.60	15.62 ± 6.49	0.947
**PVLT**			
Immediate recall	13.94 ± 7.68	12.24 ± 8.65	0.389
Short delay recall	2.83 ± 3.21	2.56 ± 3.39	0.735
Long delay recall	1.97 ± 3.00	0.97 ± 1.99	0.107
Distractor recall	2.03 ± 1.54	1.53 ± 1.38	0.161
Intrusions	10.29 ± 11.02	8.21 ± 9.92	0.413
CDR	6.10 ± 3.03	6.63 ± 3.36	0.497

*Means ± standard deviation. MMSE, Mini-Mental State Examination; PVLT, 12-Word Philadelphia Verbal Learning Test; CDR, Clinical Dementia Rating.*

### Primary Outcomes

The mean PVLT scale scores after the 2-week treatment were significantly different between the two groups [group effect, *F* = 4.43, *p* = 0.039, mean square error (MSE) = 291.51; scale effect, *F* = 234.94, *p* < 0.001, MSE = 3,888.96; group × scale interaction effect, *F* = 5.38, *p* = 0.006, MSE = 89.09]. Subsequent *post hoc* Student’s *t*-test analysis demonstrated that patients in the rTMS group scored significantly higher than those in the sham group on the PVLT total score (*t* = 2.10, *p* = 0.039) and its immediate recall subscale score (*t* = 2.38, *p* = 0.020; see Table 2).

**TABLE 2:** Scores (means ± SD) of MMSE, CDR, and PVLT at baseline and after rTMS/sham treatment in the rTMS group (*n* = 35) and sham group (*n* = 34).

	**rTMS group**			**Sham group**			**95% CI (SA vs. rA)**
	**Baseline**	**After treatment (SA)**	**95% CI**	**Baseline**	**After treatment (rA)**	**95% CI**	
**MMSE**	15.71 ± 5.60	17.17 ± 6.06**	−2.32 to −0.59	15.62 ± 6.49	16.18 ± 6.95	−1.50 to 0.38	−2.14 to 4.13
Time orientation	1.20 ± 1.51	1.57 ± 1.46*	−0.70 to −0.05	1.47 ± 1.31	1.29 ± 1.45	−0.25 to 0.60	−0.42 to 0.98
Place orientation	3.20 ± 1.47	3.40 ± 1.63	−0.60 to 0.20	3.00 ± 1.63	3.24 ± 1.83	−0.63 to 0.16	−0.67 to 1.00
Registration	2.66 ± 0.48	2.83 ± 0.45	−0.35 to 0.01	2.50 ± 0.75	2.56 ± 0.82	−0.32 to 0.20	−0.05 to 0.59
Attention and calculation	2.11 ± 1.75	2.40 ± 1.85	−0.66 to 0.09	2.38 ± 1.76	2.62 ± 2.00	−0.59 to 0.12	−1.14 to 0.71
Recall	0.51 ± 0.89	0.91 ± 0.95*	−0.76 to −0.04	0.44 ± 0.75	0.62 ± 0.89	−0.41 to 0.06	−0.15 to 0.74
Language	6.03 ± 2.09	6.06 ± 2.25	−0.41 to 0.36	5.82 ± 2.53	5.85 ± 2.43	−0.49 to 0.43	−0.92 to 1.33
Naming	1.97 ± 0.17	1.94 ± 0.24	−0.03 to 0.09	1.82 ± 0.52	1.88 ± 0.41	−0.14 to 0.02	−0.10 to 0.22
Repetition	0.54 ± 0.51	0.60 ± 0.50	−0.22 to 0.11	0.62 ± 0.49	0.53 ± 0.51	−0.09 to 0.27	−0.17 to 0.31
Three−stage command	1.89 ± 1.02	1.86 ± 1.03	−0.30 to 0.36	1.50 ± 1.13	1.62 ± 1.23	−0.42 to 0.19	−0.31 to 0.79
Reading	0.74 ± 0.44	0.77 ± 0.43	−0.09 to 0.03	0.71 ± 0.46	0.68 ± 0.47	−0.08 to 0.13	−0.12 to 0.31
Writing	0.49 ± 0.51	0.49 ± 0.51	−0.08 to 0.08	0.62 ± 0.49	0.59 ± 0.50	−0.13 to 0.19	−0.34 to 0.14
Copying	0.40 ± 0.50	0.40 ± 0.50	−0.08 to 0.08	0.56 ± 0.50	0.56 ± 0.50	−0.17 to 0.17	−0.40 to 0.08
**PVLT**	18.74 ± 12.91	24.89 ± 14.08***#	−7.93 to −4.36	15.77 ± 12.73	17.77 ± 14.04*	−3.58 to −0.42	0.36 to 13.88
Immediate recall	13.94 ± 7.68	18.23 ± 8.01***#	−5.5 to −3.07	12.24 ± 8.65	13.24 ± 9.41	−2.28 to 0.28	0.80 to 9.19
Short delay recall	2.83 ± 3.21	4.17 ± 3.57**	−2.18 to −0.51	2.56 ± 3.39	2.97 ± 3.05	−1.37 to 0.55	−0.40 to 2.80
Long delay recall	1.97 ± 3.00	2.49 ± 3.81	−1.10 to 0.07	0.97 ± 1.99	1.56 ± 2.98	−1.20 to 0.02	−0.72 to 2.57
Distractor recall	2.03 ± 1.54	1.69 ± 1.02	−0.12 to 0.81	1.53 ± 1.38	1.53 ± 1.40	−0.33 to 0.33	−0.43 to 0.74
Intrusions	10.29 ± 11.02	12.11 ± 10.34	−4.62 to 0.96	8.21 ± 9.92	8.74 ± 10.57	−2.21 to 1.15	−1.65 to 8.40
**CDR**	6.10 ± 3.03	5.96 ± 2.92	−0.14 to 0.43	6.63 ± 3.36	6.56 ± 3.45	−0.21 to 0.35	−2.15 to 0.95
Memory	1.41 ± 0.50	1.34 ± 0.55	−0.04 to 0.19	1.40 ± 0.61	1.38 ± 0.63	−0.08 to 0.11	−0.33 to 0.24
Orientation	1.18 ± 0.66	1.09 ± 0.61	−0.01 to 0.19	1.24 ± 0.62	1.25 ± 0.65	−0.09 to 0.07	−0.47 to 0.14
Judgment and problem solving	1.07 ± 0.58	1.06 ± 0.57	−0.08 to 0.11	1.19 ± 0.77	1.18 ± 0.78	−0.02 to 0.04	−0.45 to 0.21
Community affairs	1.09 ± 0.61	1.10 ± 0.55	−0.11 to 0.08	1.06 ± 0.62	1.07 ± 0.57	−0.11 to 0.08	−0.24 to 0.30
Home and hobbies	1.06 ± 0.62	1.10 ± 0.60	−0.12 to 0.03	1.22 ± 0.71	1.24 ± 0.74	−0.14 to 0.11	−0.46 to 0.19
Personal care	0.29 ± 0.63	0.27 ± 0.62	−0.03 to 0.09	0.53 ± 0.71	0.44 ± 0.66	−0.04 to 0.22	−0.49 to 0.13

*p < 0.05; **p < 0.01; ***p < 0.001 vs. baseline; #*p* < 0.05 vs. sham group. CI, confidence interval; MMSE, Mini-Mental State Examination; PVLT, 12-Word Philadelphia Verbal Learning Test; CDR, Clinical Dementia Rating.

Within the rTMS group, we found some significant improvements after the 2-week treatment, which was manifested in the PVLT total score (*t* = -6.99, *p* < 0.001) and its immediate recall (*t* = -7.19, *p* < 0.001) and short delay recall (*t* = -3.26, *p* = 0.003) subscale scores. Within the sham group, the PVLT total score (*t* = -2.57, *p* = 0.015) was significantly improved (also see Table 2).

Meanwhile, the statistical results of repeated-measures ANOVA were also consistent with the above results. Repeated-measures ANOVA also showed a significant group effect (*F* = 4.54, *p* = 0.041, MSE = 1,045.07), time effect (*F* = 53.24, *p* < 0.001, MSE = 556.07), and group × time effect (*F* = 10.33, *p* = 0.003, MSE = 142.07) on the PVLT total score, and *post hoc* analysis revealed significant improvement in the rTMS group (*F* = 48.79, *p* < 0.001), in the sham group (*F* = 6.60, *p* = 0.015), and between the two groups after treatment (*F* = 6.69, *p* = 0.014). A significant group effect (*F* = 4.47, *p* = 0.042, MSE = 466.94), time effect (*F* = 39.52, *p* < 0.001, MSE = 232.97), and group × time effect (*F* = 12.31, *p* = 0.001, MSE = 88.97) were also found on its immediate recall subscale score, and *post hoc* analysis revealed significant improvement in the rTMS group (*F* = 51.65, *p* < 0.001) and between the two groups after treatment (*F* = 7.43, *p* = 0.010). A significant time effect was found on its short delay recall subscale score (*F* = 7.88, *p* = 0.008, MSE = 25.60), and *post hoc* analysis revealed a significant improvement in the rTMS group (*F* = 10.63, *p* = 0.003). Although there was a significant time effect on the long delay recall subscale score (*F* = 7.06, *p* = 0.010, MSE = 10.48), *post hoc* analysis revealed no significant improvement in either the rTMS group (*F* = 3.19, *p* = 0.083) or sham group (*F* = 3.87, *p* = 0.058). No significant group effect (*F* = 1.26–1.31, *p* = 0.257–0.266, MSE = 3.71–256.96), time effect (*F* = 1.48–2.14, *p* = 0.149–0.228, MSE = 1.01–47.95), or group × time effect (*F* = 0.65–1.48, *p* = 0.228–0.424, MSE = 1.01–14.55) was found on other subscale scores.

### Secondary Outcomes

Within the rTMS group, we found some significant improvements after the 2-week treatment, which was manifested in the MMSE total score (*t* = -3.43, *p* = 0.002) and its time orientation (*t* = -2.33, *p* = 0.026) and recall (*z* = -2.29, *p* = 0.026) subscale scores (also see Table 2).

Meanwhile, a significant time effect was found on MMSE total score (*F* = 10.08, *p* = 0.003, MSE = 36.03), and *post hoc* analysis revealed a significant improvement in the rTMS group (*F* = 11.77, *p* = 0.002). A significant group × time effect was found on its time orientation subscale scores (*F* = 4.25, *p* = 0.047, MSE = 2.65), and *post hoc* analysis revealed a significant improvement in the rTMS group (*F* = 5.44, *p* = 0.026). Although there was a significant time effect on attention and calculation subscale score (*F* = 4.17, *p* = 0.045, MSE = 2.34), *post hoc* analysis revealed no significant improvement in either the rTMS group (*F* = 2.36, *p* = 0.134) or sham group (*F* = 1.82, *p* = 0.186). No significant group effect (*F* = 0.00–2.49, *p* = 0.119–0.984, MSE = 0.00–3.37), time effect (*F* = 0.00–2.50, *p* = 0.119–1.000, MSE = 0.00–1.63), or group × time effect (*F* = 0.00–3.09, *p* = 0.083–1.000, MSE = 0.00–0.18) was found on other subscale scores.

### Adverse Effects

Two patients in the rTMS group and one patient in the sham group did not tolerate the rTMS/sham treatment due to adverse effects. One patient in the rTMS group reported local scalp discomfort persisting for longer than 15 min after the first treatment session. The other two patients (one in the rTMS group and one in the sham group) reported feeling transient fatigue, but they could not tolerate it and asked to withdraw. There were no serious adverse effects, i.e., seizures or manic episodes.

## Discussion

After the 2-week treatment, patients in the rTMS group performed better on MMSE and PVLT scales, and these improvements were significantly greater than those in the sham group, confirming most of our two hypotheses. This study is dedicated to selecting a precision target in the left parietal cortex with the highest functional connectivity to the hippocampus as the stimulation site and has demonstrated that this novel site is remarkably effective in improving the memory of AD patients. Given the situation that there is no effective way to cure AD, preliminary but promising findings of our study have brought hope to the clinical treatment in the future.

The result that some cognition improvements were accomplished after a 2-week rTMS treatment could be supported by the finding that a 2-week rTMS treatment applied over the left lateral parietal cortex could improve cognition and modulate the functional connectivity of the brain network in AD patients (Velioglu et al., 2021). AD patients’ cognition decline might be related to disrupted integration and segregation in their large-scale brain networks (Dai and He, 2014), including the parietal memory network (Hu et al., 2019), inferring that connectivity between different brain regions was abnormally disturbed and that rTMS might strengthen residual brain tissue connectivity. Evidence showed that the functional connectivity value between the parietal cortex and hippocampus was positively correlated with associative memory improvement in healthy adults (Wang et al., 2014; Tambini et al., 2018). Our patients in the rTMS group scored significantly higher on the PVLT total score and its immediate recall subscale score. This is in line with findings that AD patients performed better on visual recognition memory after a 2-week 20 Hz rTMS treatment applied over the left lateral parietal cortex (Velioglu et al., 2021). In healthy people, transcranial direct current stimulation applied over the parietal cortex improves verbal episodic memory during the retrieval phase (Manenti et al., 2013). rTMS applied over the left lateral parietal cortex successfully increased the cortical–hippocampal connectivity and enhanced associative memory, inducing localized long-term potentiation of cortical–hippocampal networks (Wang et al., 2014). Long-term potentiation, the main form of synaptic plasticity, was considered an essential central cellular mechanism of memory and learning, and synaptic plasticity enhancement might explain rTMS efficacy in the cerebral cortex (Bliss and Collingridge, 1993; Esser et al., 2006). A study showing that synaptic plasticity induction was linked to rTMS aftereffect also supported this explanation (Hoogendam et al., 2010).

In the sham group, AD patients’ PVLT total score improved significantly compared with their baseline, which was beyond our expectation. There were some possible reasons to explain this result. One was that patients continued to take medication, including cognitive enhancers, during rTMS sessions, enhancing patients’ memory. Another could be explained by placebo response, an improvement of clinical symptoms caused by placebo treatment, or the environment in which patients received treatment. Patients’ improvements might be linked to their optimistic attitudes, goal seeking, and expected treatment outcomes (Horing et al., 2014). Furthermore, the special attention of patients’ caregivers and medical staff might also be a possible reason. Nonetheless, in the two groups, the proportions of patients who used medications were similar, reaching about 50%, and within the sham group, patients’ immediate recall, short delay recall, and long delay recall were not statistically significant after the 2-week sham treatment. Meanwhile, improved patients’ memory in the rTMS group was significantly better than that in the sham group. Therefore, we could exclude a medication effect and reasonably believe that the rTMS treatment is effective.

The rTMS treatment has the advantage of short treatment time and quick therapeutic response. Taking AD as an example, a systematic review showed that the duration of rTMS treatment varied from 1 to 18 weeks (Devi et al., 2014). After a single session of rTMS, action naming was improved immediately (Cotelli et al., 2006). And after 2–4 weeks of high-frequency rTMS, cognitive function was significantly strengthened, and this curative effect could be sustained for 2 months after treatment (Cotelli et al., 2011). Moreover, a 2-week rTMS treatment was as sufficient as longer-term treatment regarding the contribution on cognitive improvements in AD patients (Cotelli et al., 2011). The lasting time of rTMS efficacy in our study is not yet known and needs to be determined by follow-up studies.

However, there are several limitations in the current study. Firstly, there was no follow-up study on how long the effect of cognition and memory improvement could last. Secondly, we only used several neuropsychological scales to evaluate AD patients’ memory and cognition, which might not provide a comprehensive understanding. Next, we would conduct follow-up studies using neuropsychological assessments and functional magnetic resonance imaging to determine rTMS efficacy duration. Thirdly, considering AD patients’ benefits, more than half of them continued dementia-related medication at the same dosage, despite the fact that used medication presented no significant difference between the two groups.

## Conclusion

This study is a pilot study to demonstrate the precision rTMS effect over the left parietal cortex on memory and cognition improvement in AD patients, presenting a novel and promising therapy for treating memory impairment in AD patients; provides an add-on treatment for AD patients who are taking medication; and lays the foundation for our future studies on functional connectivity of brain regions and efficacy lasting time. Moreover, efforts will also be made to identify the therapeutic mechanism of AD, explore rTMS frequency and number of sessions, and further establish an optimal protocol.

## Data Availability Statement

The raw data supporting the conclusions of this article will be made available by the authors, without undue reservation.

## Ethics Statement

The studies involving human participants were reviewed and approved by the Ethics Committee of Sir Run Run Shaw Hospital, School of Medicine, Zhejiang University (No: 20170228-1). The patients/participants provided their written informed consent to participate in this study.

## Author Contributions

WC, WH, MQ, and XLi conceived the study. YJ, LX, KY, YZhang, XLv, ZZ, ZC, YZhu, LW, YS, and WC conducted the tests and collected the data. YJ, LX, and KY analyzed the data. YJ, LX, KY, YS, and WC drafted the manuscript. All authors contributed to the article and approved the submitted version.

## Conflict of Interest

The authors declare that the research was conducted in the absence of any commercial or financial relationships that could be construed as a potential conflict of interest. The reviewer TC declared a shared affiliation, with no collaboration, with one of the authors XLi to the handling editor at the time of the review.

## Footnotes

1 http://114.255.48.20/login
